# Differentiated Epithelial- and Mesenchymal-Like Phenotypes in Subcutaneous Mouse Xenografts Using Diffusion Weighted-Magnetic Resonance Imaging

**DOI:** 10.3390/ijms141121943

**Published:** 2013-11-05

**Authors:** Ya-Wen Chen, Huay-Ben Pan, Hui-Hwa Tseng, Hsiao-Chien Chu, Yu-Ting Hung, Yi-Chen Yen, Chen-Pin Chou

**Affiliations:** 1 National Institute of Cancer Research, National Health Research Institutes, Miaoli 350, Taiwan; E-Mails: ywc@nhri.org.tw (Y.-W.C.); 980736@nhri.org.tw (H.-C.C.); 940425@nhri.org.tw (Y.-C.Y.); 2 Graduate Institute of Basic Medical Science, China Medical University, Taichung 404, Taiwan; 3 Department of Radiology, Kaohsiung Veterans General Hospital, Kaohsiung 813, Taiwan; E-Mails: hbpan@vghks.gov.tw (H.-B.P.); fatsesame@gmail.com (Y.-T.H.); 4 Department of Medical Imaging and Radiological Sciences, I-Shou University, Kaohsiung 824, Taiwan; 5 School of Medicine, National Yang-Ming University, Taipei 112, Taiwan; E-Mail: hhtseng@vghks.gov.tw; 6 Department of Pathology, Kaohsiung Veterans General Hospital, Kaohsiung 813, Taiwan; 7 Department of Medical Laboratory Sciences and Biotechnology, Fooyin University, Kaohsiung 807, Taiwan

**Keywords:** diffusion-weighted magnetic resonance imaging, epithelial, mesenchymal, apparent diffusion coefficient, mouse xenografts

## Abstract

Epithelial-mesenchymal transition (EMT) is important for tumor metastasis. Detection of EMT protein expression and observation of morphological changes are commonly used to identify EMT. Diffusion-weighted magnetic resonance imaging (DW-MRI) and measuring apparent diffusion coefficient (ADC) values are noninvasive techniques for characterizing tumor microenvironments. We investigated the difference in ADC values between epithelial- and mesenchymal-like subcutaneous mouse xenografted tumors using DW-MRI. Epithelial-like MM189 PB-Klf4 and BL322 PB-Klf4 cells were generated from tumor suppressive Kruppel-like factor 4 (Klf4)-expressing mesenchymal-like MM189 and BL322 cells. The ADC values of xenografted tumors from epithelial-like MM189 PB-Klf4 and BL322 PB-Klf4 were significantly lower than those from their mesenchymal-like counterparts (*p* < 0.05 and *p* < 0.01, respectively). Our results suggested that DW-MRI is a potential tool for observing mesenchymal- or epithelial-like characteristics of subcutaneous xenografted tumors.

## 1. Introduction

Excessive cell proliferation and angiogenesis are hallmarks of the initiation and early growth of primary epithelial cancers [1]. The subsequent acquisition of invasiveness is thought to be the onset of late-stage tumorigenesis. The activation of an epithelial-mesenchymal transition (EMT) process with an important role in invasion and metastasis has been proposed as the critical mechanism for acquisition of malignant phenotypes by epithelial cancer cells [2–4]. EMT is also a key step during embryonic morphogenesis, heart development, chronic degenerative fibrosis, and cancer progression [5]. During EMT, epithelial cancer cells lose many of their epithelial characteristics, acquire mesenchymal cell properties, show reduced intercellular adhesion, display increased motility, and promote tumor metastasis [4,6]. Low E-cadherin, high vimentin, and *N*-cadherin expression are traditional markers used to identify cells that have undergone EMT [7,8].

Optic imaging has some limitations in detecting deep-seated tumors and targeting tumors with poor spatial resolution. Diffusion-weighted magnetic resonance imaging (DW-MRI) can facilitate the investigation of subtle changes during tumor progression and early responses to therapy in both clinical practice and animal models. The advantages of DW-MRI include the elimination of intravenous contrast agents for tumor enhancement and less total scanning time for the detection of both primary tumors and metastases [9,10]. DW-MRI is a technique sensitized to the diffusive characteristics of water molecules that allows noninvasive *in vivo* detection and provides information about the functional status of cells in the tissue microenvironment [11–15]. The microscopic mobility of water, classically called Brownian motion, is related to thermal agitation and highly influenced by restrictive barriers (cell membranes, organelles, macromolecules, and extracellular and intracellular space) [16]. The apparent diffusion coefficient (ADC), a measurable DW-MRI parameter of the Brownian motion of water, is related to cellular density, tumor grade, tumor subtype, Ki-67 index, and cell apoptosis [17,18]. As tumor cells proliferate, they typically crowd a given area and hinder the diffusion of water, resulting in a lower ADC value; hence, DW-MRI demonstrates hyperintensity and low ADC values in malignant tumors compared with those in normal tissues [11–15]. DW-MRI may be an effective molecular imaging tool for assessing the disorganized characteristics of tumors *in vivo* [19].

We investigated the relationship between ADC values and epithelial-/mesenchymal-like phenotypes using mouse xenografted tumor models generated from established liver cancer cells. These mesenchymal-like cells have been demonstrated to display differentiated expression of EMT-related proteins and higher capabilities for migration, invasion, and lung metastasis compared with those of epithelial-like cells [20–22]. Periodically rotated overlapping parallel lines with enhanced reconstruction (PROPELLER) DW-MRI [23] and echo planar imaging (EPI) DW-MRI techniques [17] were conducted on mice with subcutaneous tumors displaying epithelial- or mesenchymal-like phenotypes. This study assessed the relationship between ADC values of DW-MRI and epithelial- and mesenchymal-like phenotypes of mouse xenografted tumors, thereby enabling the noninvasive monitoring of epithelial- or mesenchymal-like tumors in mice.

## 2. Results

### 2.1. Epithelial- or Mesenchymal-Like Xenografted Tumors Generated from Kruppel-Like Factor 4 (Klf4) or Vector Control Expressing Mesenchymal-Like Cells

**Figure 1:**
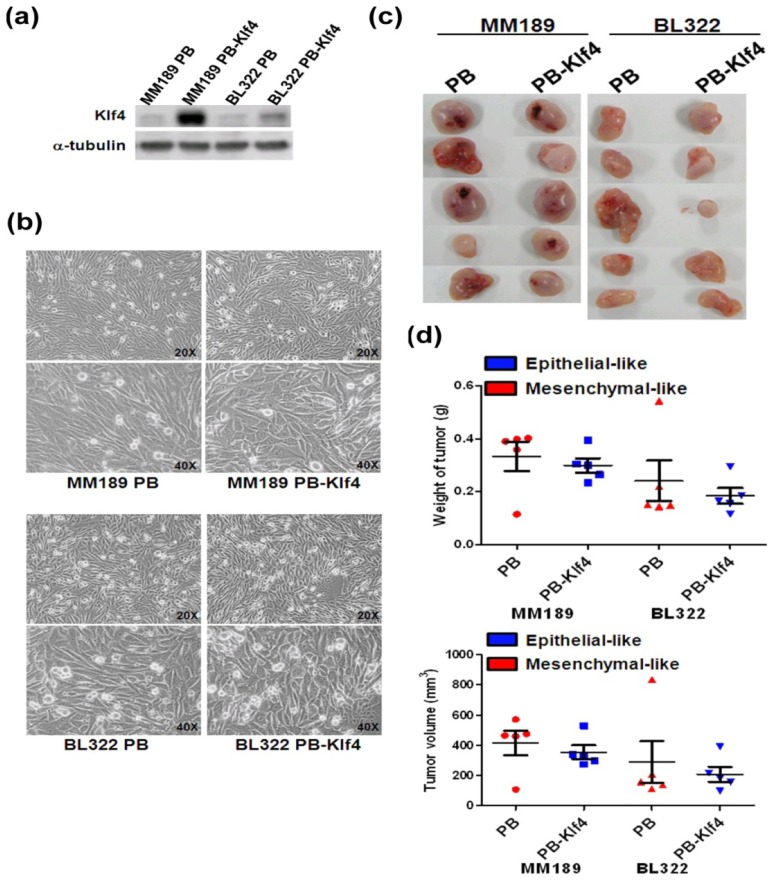
Comparison of tumor weight and volume in epithelial- and mesenchymal-like xenografted tumors. (**a**) Kruppel-like factor 4 (Klf4) and α-tubulin protein levels were detected in MM189 and BL322 with ectopic Klf4 expression (MM189 PB-Klf4/BL322 PB-Klf4) and their corresponding vector controls (MM189 PB/BL322 PB) on immunoblot assay. α-tubulin served as a loading control; (**b**) Phase contrast images of mesenchymal-like MM189 PB/BL322 PB and epithelial-like MM189 PB-Klf4/BL322 PB-Klf4 grown for 3 days on collagen-coated dishes were taken under a light field microscope with 200× and 400× magnifications; (**c**) Xenografted tumors generated from mesenchymal-like MM189 PB/BL322 PB (left flanks of animals) and epithelial-like MM189 PB-Klf4/BL322 PB-Klf4 cells (right flanks of animals); and (**d**) Quantification of xenografted tumor weight (**upper panel**) and volume (**lower panel**).

Our previous study suggested that tumor-suppressive Klf4 undergoes mesenchymal epithelial transition (MET) and reduces the capability for migration, invasion, and lung metastasis in mesenchymal-like cells [20]. To regulate epithelial- or mesenchymal-like phenotypes, we infected two mesenchymal-like liver cancer cell lines, MM189 and BL322, with a retroviral vector encoding mouse Klf4 (MM189 PB-Klf4 and BL322 PB-Klf4) or empty vector (MM189 PB and BL322 PB) and determined ectopic Klf4 expression with immunoblot assay (Figure 1a). Ectopic expression of Klf4 changed the morphology of mesenchymal-like MM189 PB and BL322 PB cells to a more epithelial-like phenotype, confirmed by observation of cell morphology with a light field microscope (Figure 1b). The subcutaneous flank tumors generated from mesenchymal-like MM189 PB cells and epithelial-like MM189 PB-Klf4 cells showed similar tumor volume and weight (Figure 1c,d). The MM189 PB tumors showed an average size of 0.333 ± 0.055 g compared with MM189 PB-Klf4 tumors, which had an average size of 0.3 ± 0.027 g (see Figure 1c). Similar results were obtained in mesenchymal-like BL322 cells. The BL322 PB tumors, with an average size of 0.241 ± 0.077 g, demonstrated tumor weight and volume similar to those of BL322 PB-Klf4 tumors, which had an average size of 0.184 ± 0.03 g (see Figure 1c,d).

### 2.2. Mesenchymal-Like Tumors Have Higher ADC Values than Those of Corresponding Epithelial-Like Tumors

**Figure 2:**
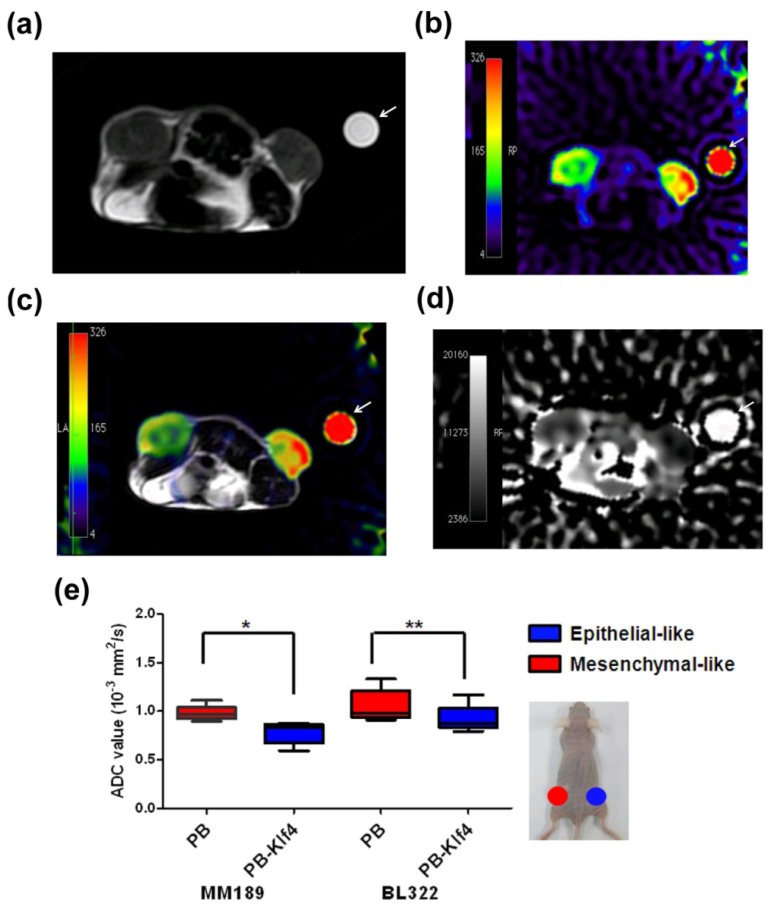
Application of periodically rotated overlapping parallel lines with enhanced reconstruction diffusion-weighted magnetic resonance imaging (DW-MRI) for *in vivo* visualization of epithelial- and mesenchymal-like tumors. (**a**) A tumor-bearing mouse carrying a left flank MM189 PB (mesenchymal-like) tumor and a right flank MM189 PB-Klf4 (epithelial-like) tumor. T2-weighted MRI shows no apparent difference in signal intensity between the bilateral flank tumors. A water phantom (white arrow) was placed within the field of view as a measuring reference; (**b**) Color-coded DW-MRI (*b* = 750 s/mm^2^) was used to visualize the signal intensity of bilateral flank tumors. Relative hyperintensity of right flank tumors (**red**) compared with left flank tumors (**green**) was observed; (**c)** Color-coded map of DW-MRI was layered over the gray-scaled anatomic T2-weighted images; (**d**) Apparent diffusion coefficient (ADC) map shows that the right flank MM189 PB-KLF4 tumor was hypointense relative to left flank MM189 PB tumor; and (**e**) Both epithelial-like MM189 PB-Klf4 and BL322 PB-Klf4 tumors showed significantly lower ADC values compared with that of the corresponding mesenchymal-like controls MM189 PB and BL322 PB, respectively. *p < 0.05; **p< 0.01.

On conventional T2-weighted MRI images, the size of subcutaneous flank tumors generated from mesenchymal-like MM189 PB and BL322 PB cells was similar to that of epithelial-like MM189 PB-Klf4 and BL322 PB-Klf4 cells (Figure 2a). No significant difference in signal intensity on T2-weighted images was seen between bilateral flank tumors in all animals (*p* > 0.05). By contrast, DW-MRI demonstrated that compared with epithelial-like MM189 PB-Klf4 tumors (*n* = 5), mesenchymal-like MM189 PB xenografted tumors were relatively hypointense on DW-MRI (*b* = 750 or 1000 s/mm^2^), relatively hyperintense on an ADC map, and had higher ADC values (Figure 2b–d). The ADC values of mesenchymal-like MM189 PB and epithelial-like MM189 PB-Klf4 tumors were 0.979 ± 0.035 × 10^−3^ mm^2^/s and 0.781 ± 0.051 × 10^−3^ mm^2^/s, respectively (*n* = 5, *p* < 0.05; Figure 2e). Similar results were confirmed in the mesenchymal-like BL322 cancer cell line. The ADC values of mesenchymal-like BL322 PB and epithelial-like BL322 PB-Klf4 tumors were 1.053 ± 0.075 and 0.922 ± 0.065 × 10^−3^ mm^2^/s, respectively (*n* = 5, *p* < 0.01; see Figure 2e). Our results indicated that epithelial-like xenografted tumors had lower ADC values compared with those of mesenchymal-like tumors.

### 2.3. Epithelial- or Mesenchymal-Like Phenotypes in Two Cancer Cell Lines

**Figure 3:**
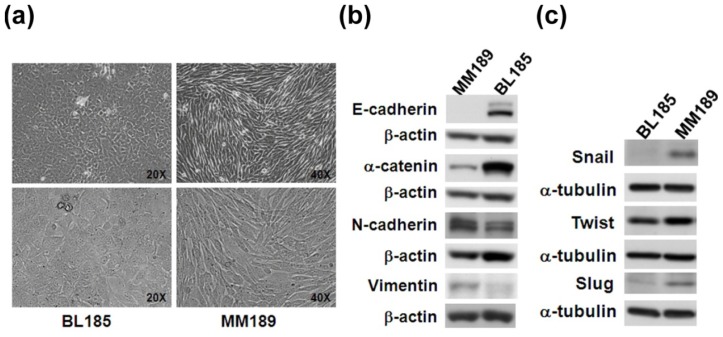
Cell morphology and immunoblot assay for epithelial-mesenchymal transition (EMT) protein markers in BL185 and MM189 cells. (**a**) Phase contrast images of epithelial-like BL185 and mesenchymal-like MM189 grown for 3 days on collagen-coated dishes were taken under a light field microscope at 200× and 400× magnifications; (**b**) Immunoblot analysis of epithelial and mesenchymal proteins including E-cadherin, α-catenin, *N*-cadherin, and Vimentin in BL185 and MM189 cells. β-actin served as a loading control; and (**c**) Immunoblot analysis of EMT-related transcription factors including Twist, Snail, and Slug in BL185 and MM189 cells. α-tubulin served as a loading control.

To determine whether the ADC difference can be applied in different cancer cell lines with epithelial-/mesenchymal-like phenotypes, we used two murine liver cancer cells, BL185 and MM189, which display different migration and invasion activities *in vitro* [21]. The MM189 cells, with high migration and invasion activities on transwell assay, showed a spindle-like, mesenchymal-like morphology. The BL185 cells, with low migration and invasion activities, showed an epithelial-like morphology after culturing (Figure 3a). Western blot showed that BL185 cells expressed epithelial proteins such as E-cadherin and α-catenin at levels higher than those in MM189 cells. MM189 cells with a mesenchymal-like morphology expressed mesenchymal proteins such as *N*-cadherin and Vimentin at higher levels than those in BL185 cells (Figure 3b). Western blot also demonstrated that BL185 cells expressed lower levels of EMT-related transcription factors such as Snail and Slug than those in MM189 cells (Figure 3c). The results indicated that BL185 and MM189 cells displayed epithelial- and mesenchymal-like characteristics, respectively.

### 2.4. Diverse ADC Values in Epithelial- or Mesenchymal-Like Xenografted Tumors Generated from Epithelial- or Mesenchymal-Like Cancer Cell Lines

**Figure 4:**
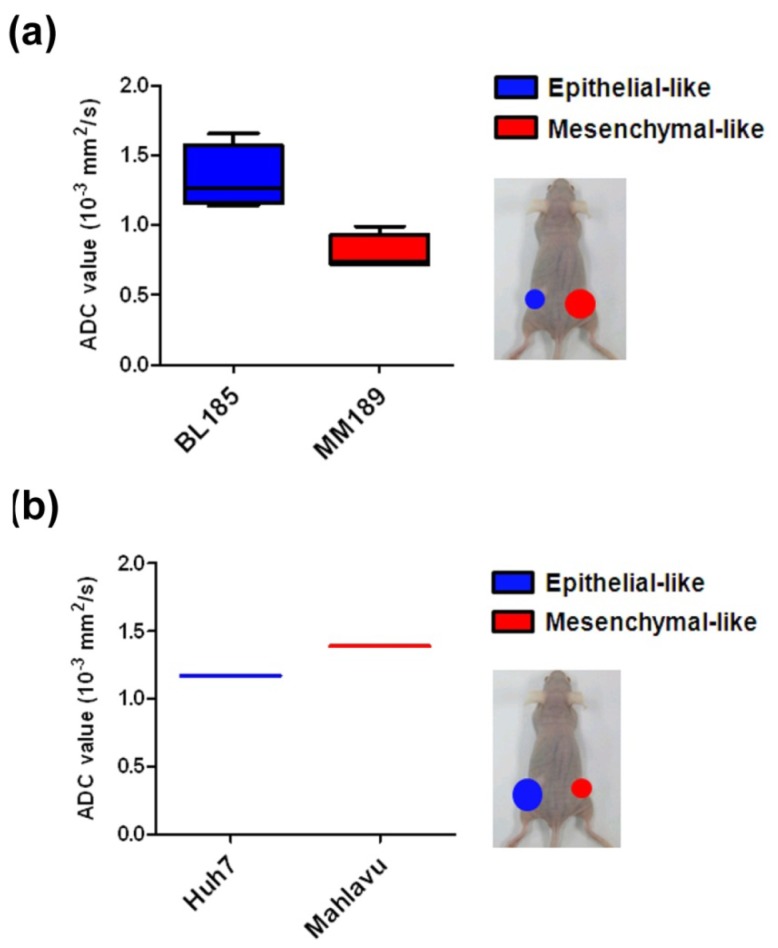
Comparison of ADC values in xenografted tumors from two different cell lines. (**a**) BL185 and MM189 subcutaneous tumors were generated from different numbers of injected cells. MM189 tumors showed significantly lower ADC values than those in BL185 tumors (*n* = 4); and (**b**) Huh7 and Mahlavu subcutaneous tumors were generated from equal numbers of injected cells. The Mahlavu tumor demonstrated an ADC value higher than that in the Huh7 tumor (*n* = 1).

The sizes of subcutaneous flank tumors generated from MM189 cells with mesenchymal-like phenotypes were larger than those from BL185 cells with epithelial-like characteristics when equal cell numbers were inoculated into the flanks of immune-compromised mice. To reduce the effect of the different tumor sizes, a high number of BL185 (10^6^) and a low number of MM189 (10^5^) cells were simultaneously injected into the flanks of mice. Subcutaneous MM189 tumors showed ADC values significantly lower than those of BL185 tumors (*n* = 4). The ADC values of MM189 and BL185 subcutaneous tumors were 0.80 ± 0.07 and 1.33 ± 0.01 × 10^−3^ mm^2^/s, respectively (Figure 4a). Additionally, we used two human liver cancer cell lines, Huh7 and Mahlavu, which show epithelial- and mesenchymal-like phenotypes, respectively [22]. ADC values of Mahlavu xenografted tumors were higher than those of Huh7 tumors. The ADC values of Huh7 and Mahlavu subcutaneous tumors were 1.17 and 1.39 × 10^−3^ mm^2^/s, respectively (Figure 4b). Collectively, these variable results might indicate that ADC values are influenced by both genetic heterogeneity and epithelial-/mesenchymal-like phenotypes among different cell lines.

### 2.5. Similar Tumor Cellularity in Epithelial- or Mesenchymal-Like Xenografted Tumors

**Figure 5:**
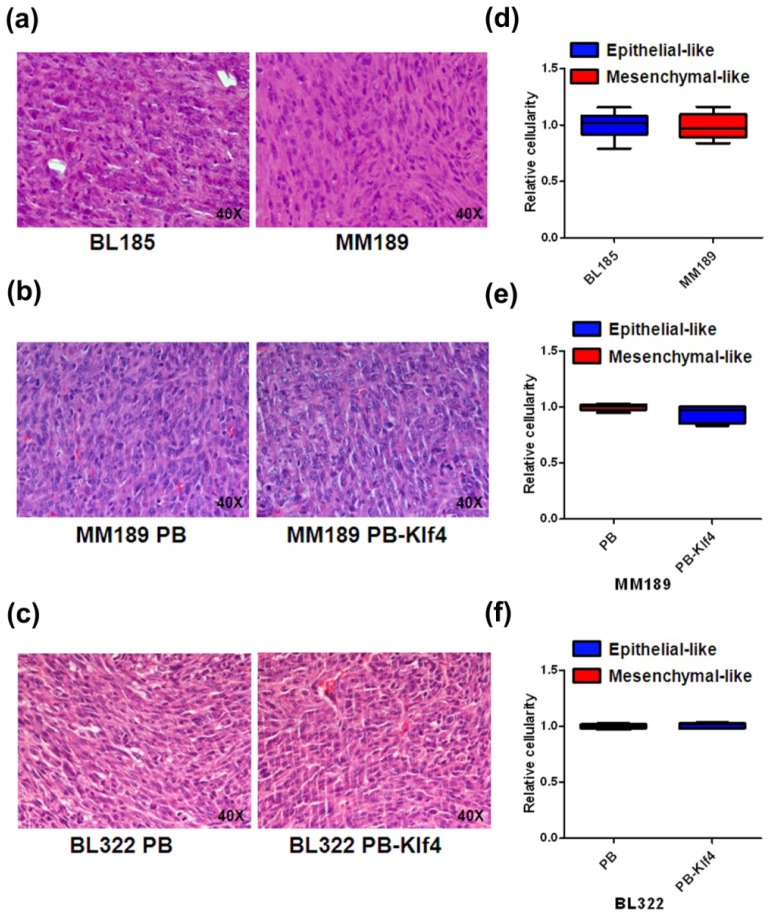
Representative hematoxylin and eosin (H&E) staining and tumor cellularity analysis. H&E-stained images (400×) of subcutaneous tumors from (**a**) BL185 and MM189; (**b**) MM189 PB and MM189 PB-Klf4; and (**c**) BL322 PB and BL322 PB-Klf4 tumors showed similar tumor cellularity. Graphs show quantitative measurement of the cellularity of (**d**) BL185 and MM189; (**e**) MM189 PB and MM189 PB-Klf4; and (**f**) BL322 PB and BL322 PB-Klf4 subcutaneous tumors.

According to previous studies, ADC value is well correlated with tumor cellularity in benign and malignant breast lesions [14]. To investigate the possibility that differentiated ADC value was caused by a change in tumor cellularity in the compared subcutaneous tumors, we stained tissue sections of excised tumors with hematoxylin and eosin (Figure 5a–c). To measure tumor cellularity, we measured the areas of all nuclei in the photographic fields using ImageJ software. Similar tumor cellularity was found among subcutaneous tumors generated form BL185 and MM189 cells (Figure 5d), MM189 PB and MM189 PB-Klf4 cells (Figure 5e), and BL322 PB and BL322 PB-Klf4 cells (Figure 5f).

## 3. Discussion

EMT, a key step in transmigration and tumor metastasis, plays an important role in clinical prognosis. In the invasion processes of primary tumors, mesenchymal-like tumor cells may have the potential to escape from the tumor mass. In primary tumors, most cells have epithelial-like phenotypes and a small number are mesenchymal-like cancer stem cells. EMT involves a multistep mechanism in which well-polarized and adhesive epithelial cells become nonpolarized, breaking through the extracellular matrix with intercellular adhesion loss mediated by cadherins at adhesive junctions and polarity markers [5]. MET, a reversal of EMT, is assumed to be important in the completion of the metastasis process [24]. Local residual or metastatic tumor cells can re-epithelialize and form a local recurrence or metastases through MET [24]. Evidence that EMT or MET is relevant to cancer progression is still under debate because of limited identification of EMT/MET *in vivo* [25]. Currently, no noninvasive imaging tool exists for the detection of EMT/MET *in vivo*. The application of DW-MRI demonstrated the capability to differentiate epithelial-/mesenchymal-like tumors on subcutaneous xenografts of immune-compromised mice.

**Figure 6:**
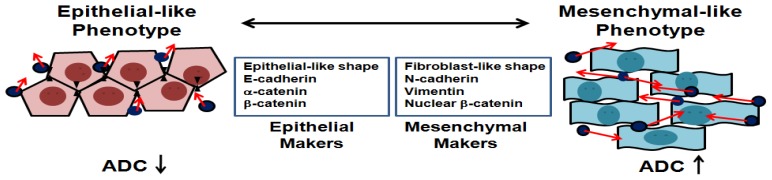
Epithelial-mesenchymal transition (EMT) is an evolutionarily conserved morphogenic process in which epithelial cells lose their epithelial characteristics and become invasive. EMT and the reverse process, mesenchymal-epithelial transition are increasingly recognized as the mechanistic basis for metastatic dissemination of epithelial tumors. Water molecules (**full circles**) and Brownian motion (**arrows**) at extracellular spaces contribute to the DW-MRI signal. Epithelial-like cells showed low ADC values, indicating that water molecules move with restriction in extracellular space. Mesenchymal-like cells with spindle-shape morphology showed higher ADC values, indicating increasing extracellular water motion.

The ADC values of DW-MRI are significantly different between mesenchymal-like and epithelial-like tumors in tumor-bearing mice (see Figure 2). In a single liver cancer cell line model, we found that the xenografted tumors from mesenchymal-like cells had lower signal intensity and higher ADC values than those from epithelial-like cells. We propose that increasing ADC value can be detected in tumor cells undergoing EMT. Conversely, metastasized tumor cells undergoing MET to establish secondary tumors demonstrate a decreasing ADC value and higher signal intensity on DW-MRI, as shown in Figure 6. E-cadherin plays an important role in maintaining cellular differentiation and normal epithelial tissue architecture, and a reduction or loss of expression is rate limiting for EMT in many carcinomas [4]. β-Catenin interacts with E-cadherin at adherens and tight junctions to maintain the epithelial phenotype. Responding to exogenous signals, β-catenin is translocated from the cell membrane to the nucleus in which it regulates gene transcription and induces EMT. The membrane expressions of E-cadherin and β-catenin complexes are often inversely correlated with tumor grade and survival stage in cancer patients, indicating favorable progression-free survival after anti-cancer therapy [8,26–30]. Higher levels of nuclear β-catenin have been detected in the invasive front of carcinomas, suggesting a more spindle-shaped/mesenchymal morphology [31]. During the transition between epithelial and mesenchymal phenotypes, water motion changes owing to loss of cell-cell contact inhibition and decreased extracellular space. Our study suggests that water diffusion is much easier when cells have less E-cadherin and β-catenin complex expression during EMT (see Figure 6). Because EMT and MET are relatively transient processes of tumor metastases, animal models using delicate cell lines offer a better opportunity to understand the complex process of clinical tumor metastases.

Decreased ADC values may correlate with several tumor characteristics, including faster tumor growth rate, increased cancer cell proliferation, and shorter doubling time for tumor growth in xenografted animal models [32]. Colorectal hepatic metastases are characterized by higher ADC values than those in normal liver parenchyma [33]. In animal models, DW-MRI showed higher sensitivity than conventional T2-weighted imaging for small liver metastases [34]. DW-MRI monitoring has been applied in animals and humans as an effective tool for determining lesion aggressiveness, monitoring response to therapy, and predicting suitable therapies [19,35,36]. DW-MRI may play an important role in the clinical management of tumor metastases.

The Warburg effect in tumors is related to high aerobic glycolysis followed by increasing glucose metabolism and lactic acid fermentation in cytosol [37,38]. The Warburg effect is different from extracellular water motion on DW-MRI. Among imaging studies, [F^18^]-fluorodeoxyglucose positron emission tomography has shown a unique capability for detecting and quantifying glucose metabolism [39]. Tumors may feature both the Warburg effect and restricted water motion. A recent study has shown that the ADC values of DW-MRI had a negative correlation with the SUVmax of [F^18^]-fluorodeoxyglucose positron emission tomography in breast cancer [39].

We found that the relationship between ADC values and the EMT phenotype of xenografts is inconsistent among various cell lines (see Figure 4). This inconsistency may be related to genomic diversity and differences in biological characteristics among cell lines. The reason that tumors have lower ADC values compared with those in normal tissues is not fully understood but is probably related to the combined effects of higher tumor cellularity, tissue disorganization in the microenvironment, higher tumor grade, and decreased extracellular water space, all leading to reduced Brownian motion of water [40]. Tumor necrosis and the vascular perfusion effect can lead to inconsistency in preference measurements of ADC values. Separation of diffusion and vascular perfusion motion of water can be accomplished using three b values [41]. Vascular perfusion, which causes intravoxel incoherent motion, is likely to occur on DW-MRI using a low b value (<200 s/mm^2^) [41]. We acquired DW-MRI with high b values (750 or 1000 s/mm^2^) to prevent interference from vascular perfusion. Increasing ADC value in malignant tumors during therapy is a promising and noninvasive method for predicting the therapeutic response of liver metastases [42].

In our study, the different ADC values of epithelial- and mesenchymal-like phenotypes did not result from tumor cellularity (see Figure 5). Some data have suggested that the mean ADCs of malignant and benign breast lesions correlate well with tumor cellularity [14]. By contrast, similar to our data, other studies have demonstrated that mean ADC does not significantly correlate with tumor cellularity, indicating that ADC value depends not only on tumor cellularity but also on histological type [43,44].

Both PROPELLER DW-MR and EPI DW-MRI can detect differences in xenografted tumors generated from cells with epithelial- and mesenchymal-like phenotypes. In our mouse experiment, PROPELLER DW-MR had better image quality and less distortion compared with that of EPI DW-MRI on a 1.5T MRI with a wrist coil. PROPELLER DW-MRI obtained excellent resolution for measuring ADC value even in small flank tumors (size <1 cm) in our study. A previous study has also demonstrated the capability of PROPELLER multishot fast spin-echo (FSE) DW-MRI to evaluate liver tumor necrosis quantitatively in rabbits [23]. PROPELLER DW-MRI, superior to DW-FSE-EPI, was well-correlated and highly concordant with histopathology in our study.

We used a common subcutaneous mouse xenografted tumor model instead of orthotopic tumor models. Observation of different tumor microenvironments in a single host is easier in the subcutaneous mouse xenografted model with bilateral flank tumors.

## 4. Materials and Methods

### 4.1. Ethics Statement

This animal study was performed in strict accordance with the recommendations in the guidelines for the care and use of laboratory animals of Kaohsiung Veterans General Hospital (Kaohsiung, Taiwan) and the National Health Research Institutes (Taipei, Taiwan). The animal experiments were conducted according to protocols approved by the Kaohsiung Veterans General Hospital with the Animal Studies Committee and Institutional Animal Care and Use Committee of the National Health Institutes and complied with the Animal Welfare Act (Protocol No: vghks-2011-A011, NHRI-IACUC-100136-A). All efforts were made to minimize suffering.

### 4.2. Cell Lines

The murine hepatocellular carcinoma cell lines BL185, MM189, and BL322 have been previously described [21,45]. Huh7 and Mahlavu were established from human hepatocellular carcinoma [46,47]. MM189 cells expressing vector control (MM189 PB), MM189 cells with ectopic Klf4 (MM189 PB-Klf4), BL222 cells expressing vector control (BL322 PB), and BL322 cells with ectopic Klf4 (BL322 PB-Klf4) were established previously [20]. All of these cells were maintained in Dulbecco’s modified Eagle’s medium (Invitrogen, San Diego, CA, USA) supplemented with 10% fetal bovine serum (Biological Industries, Kibbutz Beit Haemek, Israel). All cells were cultured at 37 °C in a 5% CO_2_ atmosphere within 3 months of resuscitation from frozen aliquots, with less than 20 passages in each experiment.

### 4.3. Cell Morphology

For cell morphology analysis, 10^5^ cells were placed into six-well tissue culture plates with collagen coating, and live cell images were taken using an inverted microscope after 3 days of culture.

### 4.4. Western Blot

Western blot was performed as described elsewhere [21]. Membranes were probed with the following primary antibodies: anti-E-cadherin (610182, BD Biosciences, Franklin Lakes, NJ, USA), anti-*N*-cadherin (610920, BD Biosciences), anti-α-catenin (610193, BD Biosciences), anti-Vimentin (MS-129-P0, Thermo Scientific, Waltham, MA, USA), anti-Twist (sc-15393, Santa Cruz, Santa Cruz, CA, USA), anti-Snail (3895, Cell Signaling, Danvers, MA, USA), anti-Slug (AP2053a, Abgent, Aachen, Germany), anti-Klf4 (sc-20691, Santa Cruz, CA, USA), anti-β-actin (sc-1615, Santa Cruz, CA, USA), and anti-α-tubulin (MS-581-P0, Thermo Scientific).

### 4.5. Xenografted Tumor Models

Cells were individually suspended in 100 μL of sterile phosphate-buffered saline and injected subcutaneously into the bilateral flanks of 5-week-old male athymic BALB/c mice (National Laboratory Animal Center, Taipei, Taiwan). Several groups of mice were established as follows: (a) 10^5^ MM189 PB and MM189 PB-Klf4 cells were subcutaneously injected into the left and right flanks of five mice, respectively; (b) 5 × 10^5^ BL322 PB and BL322 PB-Klf4 cells were subcutaneously injected into the left and right flanks of five mice, respectively; (c) 10^6^ BL185 and 10^5^ MM189 cells were injected into the left and right flanks of four mice, respectively; (d) 2 × 10^5^ Huh7 and Mahlavu cells were subcutaneously injected into the left and right flanks of one mouse, respectively. When subcutaneous tumors grew to a size of approximately 10 mm × 10 mm, all tumor-bearing mice underwent MRI examination.

### 4.6. DW-MRI

MRI of tumor-bearing mice was performed to observe the water diffusion microenvironment between epithelial tumor- and mesenchymal tumor-bearing mice *in vivo*. DW-MRI was performed with a 1.5-T clinical MRI scanner (General Electric Medical Systems, Milwaukee, WI, USA) and an 8-channel phased-array wrist coil (General Electric). Each mouse was anesthetized using 2% isoflurane (Forane, Abbott Laboratories Ltd., Queenborough, Kent, UK) and placed in the coil during MRI.

Axial images of the target-implanted tumor were obtained using a fat-saturated FSE T2-weighted sequence (TR/TE 6000/99; matrix 128 × 128; field of view [FOV] 80 × 80 mm; NEX = 8; echo train length = 32; ASSET = 2). DW-MRI examinations were carried out with FSE PROPELLER MRI or EPI MRI sequences. Total acquisition time for DW-MRI and conventional MRI was approximately 20 min.

DW-MRI was performed by PROPELLER or EPI sequence. PROPELLER DW-MRI was performed using multishot FSE sequences (TR/TE = 2100/72 ms; echo train length 16; FOV 12 cm; acquisition matrix = 128 × 128; slice thickness = 2 mm; NEX = 2; b factors = 0 and 750 s/mm^2^) at identical, contiguous axial slice positions with complete coverage of each flank tumor. PROPELLER DW-MRI was performed in three orthogonal gradient directions (*x*, *y*, and *z*) with 0 and 750 s/mm^2^ b factors. EPI DW-MRI was performed using single-shot EPI (TR/TE = 5000/78 ms) with an acquisition matrix of 96 × 96, a reconstruction matrix of 128 × 128, an FOV of 8 cm, and a slice thickness of 4 mm with two b factors (0 and 750 s/mm^2^) in three orthogonal gradient directions (*x*, *y*, and *z*). A phantom containing pure water was placed adjacent to the mice within the field of view as a reference for T2-weighted signal intensity and ADC value.

### 4.7. Post-Processing Workstation

DW-MRI data were transferred to a commercial workstation for post-processing analysis. ADC maps were reconstructed on a pixel-by-pixel basis using commercially available FuncTool software (General Electric). The radiologist initially reviewed the DW-MRI and located the flank tumors in the mice. The ADC values of the flank tumors were calculated from signal intensity changes between the b values of 0 and 750 s/mm^2^ as the negative slope of the linear regression line that best fit the points for b value *versus* ln (signal intensity). The mean ADC values of each pixel of the images and regions of interest (ROIs) were displayed as parametric ADC maps, and the ADC values of different target flank tumors were derived. Two radiologists (specialists in MRI, each with more than 10 years of experience) independently analyzed the images blinded to tumor characteristics. Between three and five oval ROIs were placed over the flank tumor on the ADC maps by each of the radiologists along the center and periphery of the solid portion of the tumor, excluding the tumor cystic lesion and hemorrhage. The final ADC values of each mouse were determined by averaging the results of all measured ROIs. If the average ADC measurements of the radiologists differed by more than 0.2 × 10^−3^ mm^2^/s, the radiologists determined the final ADC value through consensus discussion. Differences in T2-weighted images of bilateral flank tumors were determined by measuring the signal intensity on the axial image in reference to a water phantom using open-source OsiriX software (OsiriX Foundation, Geneva, Switzerland) [48]. The ROIs of T2-weighted images were drawn on areas of tumor similar to those used for DW-MRI.

### 4.8. Histology and Analysis of Tumor Cellularity

After MRI scanning, the mice were killed for histopathology study. The subcutaneous tumors were fixed in formaldehyde and processed separately for routine paraffin embedding and sectioning. Serial sections (5 μm thick) were cut on a rotary microtome, collected on polylysine-coated slides, and dried overnight at 37 °C. The sections were stained with hematoxylin and eosin and photographs were taken (original magnification 400×) at the four corners and center point of the tumor slide under light-field microscope (Nikon, Tokyo, Japan). Tumor cellularity was analyzed using Image J (National Institutes of Health, Bethesda, MD, USA) and defined as the total area of tumor cell nuclei divided by the histological section area according to the redefined threshold. The relative cellularity was calculated by dividing the cellularity of each tumor by that of its corresponding control.

### 4.9. Statistical Analysis

Mean ADC values of the bilateral flank tumors in nude mice were expressed as means, standard deviation, and ranges. Tumor weight and volume of the subcutaneous tumors were expressed as means and standard error of mean. The measured differences between bilateral flank tumors were compared using the two-tailed paired *t* test. Statistical calculation was performed using GraphPad Prism (version 5.01; GraphPad Software, San Diego, CA, USA). The value of *p* < 0.05 was considered statistically significant.

## 5. Conclusions

In subcutaneous tumors derived from single cancer cell lines, the ADC values of epithelial-like xenografted tumors are lower than those of mesenchymal-like tumors. Though this observation is inapplicable to subcutaneous tumors generated from different epithelial-/mesenchymal-like cancer cell lines, our results indicate that DW-MRI is a potential tool for observing mesenchymal- or epithelial-like characteristics of subcutaneous xenografted tumors.

## Abbreviations

TE: echo time
TR: repetition time
ADC: apparent diffusion coefficient
DW-MRI: diffusion weighted magnetic resonance imaging
EPI: echo-planar imaging
PROPELLER: periodically rotated overlapping parallel lines with enhanced reconstruction
FOV: field of view
ROI: region of interest
EMT: epithelial mesenchymal transition
MET: mesenchymal epithelial transition.
